# Optimal dose of oxytocin to improve social impairments and repetitive behaviors in autism spectrum disorders: meta-analysis and dose–response meta-analysis of randomized controlled trials

**DOI:** 10.3389/fpsyt.2024.1477076

**Published:** 2025-01-29

**Authors:** Yingying Zhang, Xiaolu Zhang, Linghong Huang

**Affiliations:** ^1^ Department of Molecular Psychology, Institute of Psychology and Education, Ulm University, Ulm, Germany; ^2^ Anhui Children’s Hospital, Pediatric Hospital Affiliated to Fudan University, Hefei, China; ^3^ The Clinical Hospital of Chengdu Brain Science Institute, Ministry of Education Key Lab for Neuroinformation, University of Electronic Science and Technology of China, Chengdu, China

**Keywords:** oxytocin, autism spectrum disorder, ASD, dose-response, meta-analysis

## Abstract

**Introduction:**

Social impairments and repetitive behaviors are at the core symptoms of autism spectrum disorder (ASD). Intranasal administration of the neuropeptide oxytocin (OXT) is a promising treatment. However, there have been inconsistencies in the effects of OXT on social impairments and repetitive behaviors.

**Methods:**

A comprehensive search in PubMed, the Cochrane Library, Embase, and Web of Science was conducted to gather randomized controlled trials (RCTs) on the efficacy of OXT in patients diagnosed with ASD up to 11/06/2024. The core outcomes were social impairments measured by total Social Responsiveness Scale (SRS) scores and repetitive behaviors measured by the Repetitive Behavior Scale (RBS).

**Results:**

This meta-analysis ultimately included 12 RCTs with 498 ASD patients. In an initial analysis, intranasal OXT showed no significant effect on social impairments. For a high dose of 48 IU per day, a beneficial effect on social impairments was found. According to the dose–response meta-analysis, the results indicated that higher doses of OXT might be more effective for social impairments. Depending on repetitive behaviors, the overall analysis showed no significant effect, while the dose over 48 IU per day revealed significant results and the dose–response meta-analysis suggested that higher doses could be more effective for repetitive behaviors.

**Discussion:**

Although these findings show no consistent beneficial effects, the results of the dose–response meta-analysis suggest that high doses of intranasal OXT per day may be more effective in ASD.

**Systematic review registration:**

https://www.crd.york.ac.uk/prospero, identifier CRD42024567213.

## 1 Introduction

Autism spectrum disorder (ASD), as a neurodevelopmental condition, is characterized by core symptoms including challenges in social communication and repetitive behaviors (1). During early development in growing fetuses and very young children, ASD is a common early-onset condition. According to the reports of the Autism and Developmental Disabilities Monitoring (ADDM) Network, the overall prevalence of ASD was 23.0 per 1,000, of which the prevalence was 4.2 times higher in boys than in girls (2). In addition, psychiatric comorbidities including anxiety, depression, attention-deficit/hyperactivity disorder, and obsessive-compulsive disorder have been observed in approximately 75% of patients with ASD (3). The pathology is complex and involves genetic and epigenetic mutations that are influenced by specific interactions of transcription factors and chromatin remodeling processes that take place in specific neurons of the brain (4). At the present time, there are no first-line medications available for the core social symptoms of ASD such as social impairments and repetitive behaviors. Taken together, it is important to understand more about how ASD works and to develop treatments to effectively combat the spread of the disorder.

Accumulating evidence showed that the hypothalamic neuropeptide oxytocin (OXT) is emerging as a promising therapeutic target (5). OXT is a peptide that has multiple functions in the body, both as a peripherally hormone and as a central neurotransmitter (6). Synthetic OT has been used to assist in childbirth for decades, and intranasal use of synthetic OXT has become the preferred way for studying its social cognitive effects among ASD children and adolescents (7–9). OXT plays a crucial role in the involvement of reproductive processes (i.e., parturition and lactation) (10) and has an important role in the socio-emotional processes that occur during social interactions. Numerous studies have been conducted to investigate the therapeutic effects of intranasal OXT in ASD, but the results have not been consistent. For instance, Yamasue et al. (2020) found that a 6-week course of intranasal OXT could improve repetitive behaviors measured by the Autism Diagnostic Observation Schedule (ADOS) and increase the gaze fixation on social regions (11). Moreover, OXT administration could also improve the social abilities of individuals with ASD and show positive effects in the reciprocal communication domain (12, 13). Meanwhile, a recent multilevel meta-analysis indicated the beneficial efficacity on social impairments of OXT (14). In comparison with the well-established beneficial effects of OXT in ASD, a variety of research reported failed or limited therapeutic effects. Likewise, Huang et al. (2021) did not observe any improvement in ASD symptoms, despite caregivers of individuals with ASD reporting significant improvements; these reports were influenced by their belief in the efficacy of the treatment for their children after OXT administration (14).

Emerging literature highlights the effects of multi-dosages such as 24 international units (IU), 32 IU, and 48 IU on improving ASD symptoms (i.e., social impairments and repetitive behaviors). Evidence has shown that the dosage of intranasal OXT might yield different effects. A recent study has reported that both excessively high and low doses of OXT may fail to demonstrate the therapeutic effects in ASD, with an inverted U-shaped dose–response curve, with the peak therapeutic effect occurring at a lower dose than expected based on earlier studies in ASD (15). For instance, Quintana et al. (2017) found that the optimal effect on the response of the amygdala to emotional faces was 8 IU delivered via the nebulizer, whereas the majority of studies reported a similar effect at a dose of 24 IU (16). In addition, the dose of OXT was found to be a significant factor in social outcomes in a recent meta-analysis by Audunsdottir et al. (2024) (17). Therefore, it is important to investigate the dose–response relationship, which is key to determining the optimal doses of OXT to improve ASD symptoms. In addition, the effects of OXT are also known to be sexually dimorphic (18, 19). The role of OXT in social behavior in both sexes often demonstrate a robust sex-specific modulation of social behavior by OXT systems in a variety of rodent species and humans. For instance, OXT impaired women’s ability to accurately perceive emotions but not men’s ability (20), whereas intranasal OXT impaired recognition memory of neutral and happy faces in men but not in women (21). These studies highlight the importance of possible sex differences of OXT effects in ASD.

Based on the above consideration, the aim of this meta-analysis, using novel meta-analytic techniques [i.e., dose–response meta-analysis (22, 23)], is to assess the effect of OXT treatment relative to placebo and to address the optimal dose of OXT to improve social impairments and repetitive behaviors. The dose–response meta-analysis plays a crucial role in examining the relationship between independent variables (e.g., OXT dosages) and disorder outcomes (e.g., social impairments and repetitive behaviors), which can provide a preliminary estimation for the effect of OXT administration on social impairments and repetitive behaviors in ASD.

## 2 Methods

This meta-analysis was preregistered in the International Prospective Register of Systematic Reviews (PROSPERO, CRD42024567213) and adhered to the Preferred Reporting Items for Systematic Reviews and Meta-Analyses (PRISMA) checklist (details see **Supplementary Table S1**). The protocol included the title, aims, inclusion and exclusion criteria, search strategies, data extraction, results, study quality assessment, publication bias, and data analytics strategies. Patient consent or ethical approval was not necessary, as all analyses were calculated based on previous studies.

### 2.1 Search strategy

A comprehensive search in online databases in PubMed, the Cochrane Library, Embase, and Web of Science was conducted up to June 2024. Search strategies specific to the combination of Medical Subject Heading terms (MeSH, including oxytocin and ASD) and free words. The full search strategies are shown in **Supplementary Table S2**. In addition, trial registries and references of the relevant research and reviews were searched for relevant RCTs.

### 2.2 Inclusion and exclusion criteria

Inclusion criteria followed by the Participant, Intervention, Comparison, Outcome, and Study design (PICOS) guidelines (24) are as follows:

Patients: patients were diagnosed with ASD, with diagnostic criteria such as DSM criteria.

Intervention: patients in the treatment group received any dosage and frequency of OXT.

Comparison: patients in the treatment group received placebo (nasal spray included all of the same ingredients except oxytocin, or normal saline).

Outcomes: the primary outcomes were overall core symptoms measured by total Social Responsiveness Scale (SRS) scores and repetitive behaviors measured by total scores of the Repetitive Behavior Scale (RBS). SRS is a widely used rating scale that measures the severity of autistic symptomatology as a quantitative trait (25). A total score of SRS serves as an index of severity of social impairments in the autism spectrum, and higher total scores on the SRS indicate greater severity of social impairment. RBS is used to measure the breadth of repetitive behavior in ASD patients, which provides a quantitative, continuous measure of the full spectrum of repetitive behaviors (26). The total score of RBS reflects the overall severity of repetitive behaviors, with higher total scores suggesting greater severity.

Study design: only randomized clinical trials (RCTs) were included.

The following studies were excluded: 1) studies where the intervention or control group used other treatment methods; 2) animal experiments, genetic, or MRI studies; 3) meta-analyses, reviews, case reports, experimental plans, comments, letters, editorials, conference papers, etc.; 4) studies with missing data or severe errors; 5) duplicate publications; 6) studies where the full text was not available.

### 2.3 Study selection and data extraction

The retrieved literature was imported into EndNote. Based on the inclusion and exclusion criteria, two researchers [Xiaolu Zhang (X.L.Z) and Linghong Huang (L.H.H)] independently screened the titles and abstracts, followed by a full-text reading for further screening. Any divergences in the literature were resolved by discussing or consulting with a third researcher [Yingying Zhang (Y.Y.Z)] for reassessment. The two researchers (X.L.Z and L.H.H) independently extracted data from the included studies using Excel 2016, including the first author, year of publication, diagnostic criteria, details of randomization and blinding, sample size, age, intervention and control treatment, efficacy evaluation criteria, and endpoint measures.

### 2.4 Quality assessment

The Cochrane risk of bias tool (RoB2.0) (27) was used to assess the qualities of the included studies. It consists of five aspects: bias arising from the randomization process, bias due to deviations from the intended interventions, bias due to missing endpoint data, bias in the measurement of the endpoints, and bias in the selection of the reported endpoints. The overall quality was rated into three categories: low risk of bias, some concerns of bias, or high risk of bias according to the items mentioned above. For a study to be rated at overall low risk of bias, all of the domains needed to be rated at low risk of bias. The study was classified as some concerns in one aspect was rated as some concerns of bias. The study is judged to be at high risk of bias in at least one domain for a high risk of bias, or the study is judged to have some concerns for multiple domains in a way that substantially lowers confidence in the result (27). For each study, two researchers (X.L.Z and L.H.H) independently judged each of the five aspects. Disagreements were resolved through discussion or consultation with a third researcher (Y.Y.Z).

### 2.5 Statistical analysis

All data analyses were calculated with Stata software (version 16, Stata Corporation, College Station, TX, USA). The standardized mean difference (SMD) and 95% confidence interval (CI) of the changes (between baseline and posttreatment of OXT administration) in the treatment group relative to the control group was calculated as the effect size. Regarding different dosages and frequencies of OXT, OXT administration was recalculated with the standard: the average dosage administrated per day to pool the results. Since doses, frequencies, and treatment duration were quite heterogeneous among the included studies, a random-effect (DerSimonian and Laird) model was adopted. The I^2^ value was used to assess the heterogeneity among the included studies. A sensitivity analysis (the “one study removed” method) was also performed to determine the effect of each trial on the pooled effect size (28). Moreover, the predefined subgroup analyses were conducted depending on different dosages of OXT administrated. Examined asymmetry in the funnel plots and Egger’s test (29) were assessed for publication bias.

In addition, following the one-stage approach (22), the dose–response meta-analyses were conducted considering the correlation between the ranged OXT dosage per day, treatment duration, and a set of mean differences of core symptoms. SMD and its 95% CI for each 1 IU/day increase in OXT administration in each RCT were calculated with the dose–response meta-analysis using the method introduced by Crippa and Orsini (2016) (22). This method requires the number of participants in each research arm, the administration dose, and the mean and standard deviation of change across the study in each research. Trial-specific means and standard errors of changes in outcome indicators for each 1 IU per day (IU/day) increase in OXT were combined using the DerSimonian and Laird random-effect model (30). A rigorous cubic spine model with three nodes (10th, 50th, and 90th percentiles) of the total dose distribution was used to assess the dose–response relationship. A two-tailed p-value < 0.05 was considered statistically significant.

## 3 Results

### 3.1 Literature screening results

**Figure 1:**
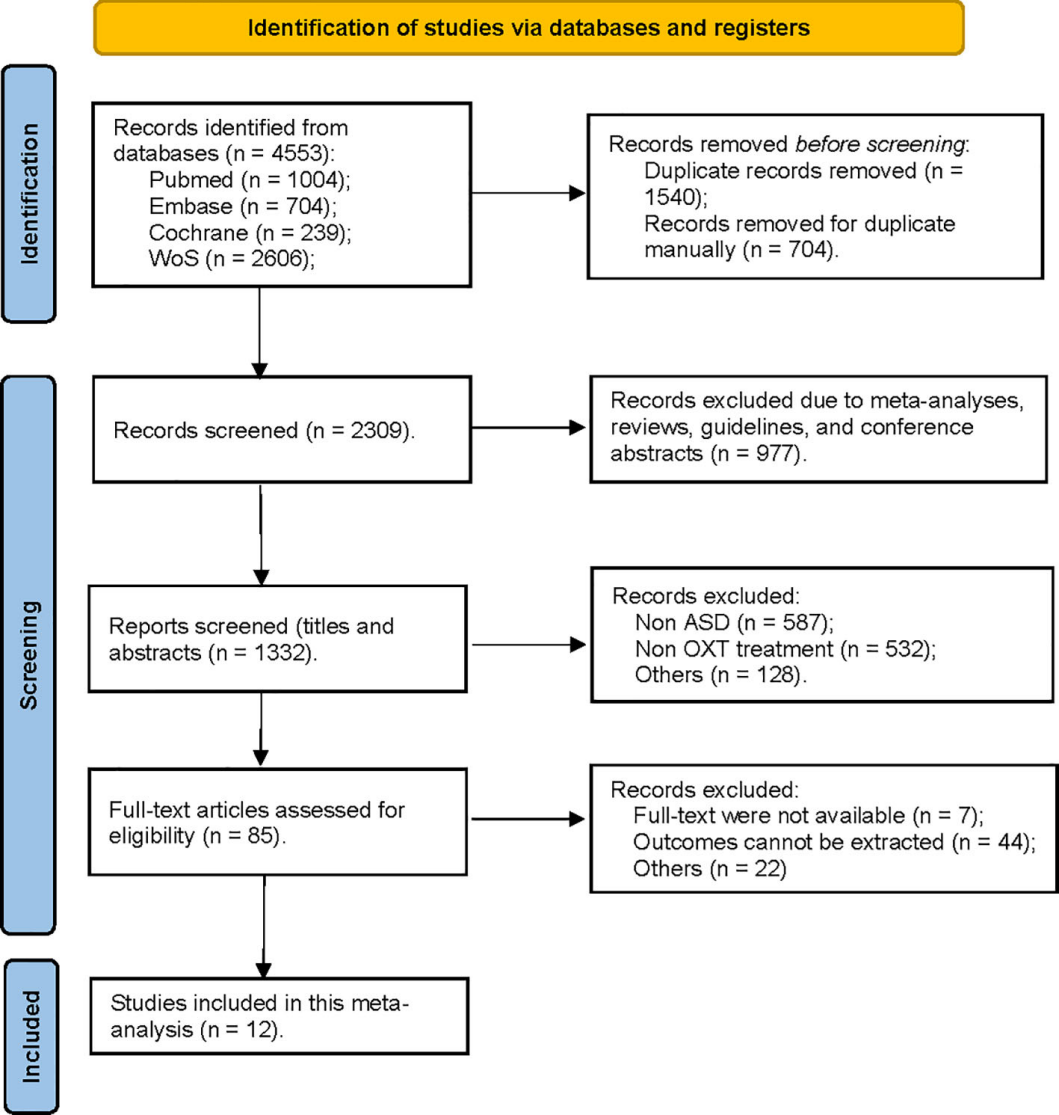
Flowchart for the search strategy qualifying studies for OXT administration and ASD in the current meta-analysis.

The initial research yielded 4,553 references. After removing duplicates automatically, the titles and abstracts of the remaining 2,309 references were screened, and then another 977 references were excluded following a screening for potential studies (reasons for exclusion included meta-analyses, reviews, guidelines, and conference abstracts). The remaining 1,332 references with titles/abstracts were further screened, and 1,247 were excluded because of not meeting the eligibility criteria. The last 85 studies were again further assessed for eligibility by examining their full texts, and 73 studies were excluded (no full texts, lacked data, or data cannot be merged). Ultimately, 12 studies were included in this meta-analysis (details see **Figure 1**).

### 3.2 Basic characteristics of the included studies

The characteristics of each included study are presented in **Table 1**. A total of 12 RCTs (13, 31–41) were included. Overall, 498 ASD patients enrolled in the 12 RCTs, with sample size ranging from 18 (40) to 87 (35), and mean ages ranging from 5.0 years old (37) to 33.4 years old (31). There were 252 patients who received intranasal OXT treatment, and the remaining 246 patients received a placebo control. Of the total sample, most patients were diagnosed with ASD with DSM-IV (31–33, 36, 38, 39), and some patients were diagnosed with ASD with DSM-V (35, 37, 38, 41), and the remaining patients were diagnosed with ASD with DSM-IV and DSM-V (13, 40). Among the included studies, five were conducted in the USA (13, 31, 33, 40, 41), three in Australia (32, 35, 36), two in Belgium (38, 39), one in the Netherlands (34), and one in China (37). Depending on the doses and treatment duration in the included studies, two studies administered 24 IU with twice-daily lasting 6 weeks (31, 37), one study administered 24 IU with twice-daily lasting 4 weeks (13), and one study administered 24 IU with once-daily lasting 4 weeks (39). One study administered 12 IU with twice-daily lasting 4 weeks (38), and one administered 32 IU with twice-daily lasting 12 weeks (35). The remaining studies administered a range of doses from once-daily or twice daily of 3 IU to 40 IU, and a range of treatment duration from 0.57 weeks (4 days) to 12 weeks (32–34, 36, 40, 41). In terms of outcomes, 11 studies (13, 31–40) examined the effects of OXT on social impairments, and 6 studies (31, 32, 34, 36, 37, 41) examined the effects of OXT on repetitive behaviors in ASD. Depending on the control group settings, patients received nasal spray including all of the same ingredients except oxytocin (13, 31–37) or normal saline (38–41) in identical bottles and labels. All the included studies were careful to avoid placebo effects by having the same experimenter giving instructions to patients. In particular, two studies used double-blind, crossover design trials where subjects received both OXT and PLC treatments to avoid placebo effects (32, 37).

**Table 1:** Basic characteristics of the included studies.

Studies	Countries	Diagnosis	Sample (N)		Male (N)		Age (M)		Dose (IU)	Duration (W)	Frequency	Outcomes
			OXT	PLC	OXT	PLC	OXT	PLC				
Anagnostou et al., 2012 (31)	USA	DSM-IV	10	9	9	7	33.8	32.9	24	6	Twice-daily	SRS, RBS
Yatawara et al., 2016 (32)	Australia	DSM-IV	15	16	14	13	5.7	6.7	3-24	5	Twice-daily	SRS, RBS
Dadds et al., 2014 (33)	USA	DSM-IV	19	20	19	20	11.8	10.7	12-24	0.57	once-daily	SRS
Damen et al., 2021 (34)	Netherlands	/	13	13	7	6	7.3	7.7	16-40	12	Twice-daily	SRS, RBS
Guastella et al., 2023 (35)	Australia	DSM-V	45	42	39	35	7.4	7.1	32	12	Twice-daily	SRS
Guastella et al., 2015 (36)	Australia	DSM-IV	26	24	26	24	13.9	14.0	18-24	8	Twice-daily	SRS, RBS
Le et al., 2022 (37)	China	DSM-V	21	20	20	18	5.1	4.9	24	6	Twice-daily	SRS, RBS
Parker et al., 2017 (13)	USA	DSM-IV/V	17	18	13	14	9.4	8.1	24	4	Twice-daily	SRS
Daniels et al., 2023 (38)	Belgium	DSM-V	38	39	30	31	10.5	10.4	12	4	Twice-daily	SRS
Bernaerts et al., 2020 (39)	Belgium	DSM-IV	22	18	22	18	25.0	24.0	24	4	once-daily	SRS
Kong et al., 2021 (40)	USA	DSM-IV/V	18	17	15	11	9.9	10.7	4-32	4	Twice-daily	SRS
Fastman et al., 2021 (41)	USA	DSM-V	8	10	4	5	6.8	9.8	12-24	12	Twice-daily	RBS

DSM-IV, Diagnostic and Statistical Manual of Mental Disorders (4th edition); DSM-V, Diagnostic and Statistical Manual of Mental Disorders (5th edition); N, numbers; M, mean; IU, international units; W, weeks; SRS, the caregiver-rated Social Responsiveness Scale; RBS, the Repetitive Behavior Scale.

### 3.3 Risk of bias assessment

The risk of bias for the included RCTs was evaluated using the Cochrane Risk of Bias tool, and the findings are presented in **Table 2**. Among the included studies, two studies showed some concerns about the bias arising from the randomization process (33, 36), one study showed some concerns about the bias due to the measurement of the outcome (32), and one study showed some concerns about the bias in the selection of the reported result (38). The remaining eight studies were at low risk of detection bias due to being blinded to assessors.

**Table 2:** The results of risk of bias assessment.

Studies	①	②	③	④	⑤	Overall
Anagnostou et al., 2012 (31)	Low	Low	Low	Low	Low	Low
Yatawara et al., 2016 (32)	Low	Low	Low	Some concerns	Low	Some concerns
Dadds et al., 2014 (33)	Some concerns	Low	Low	Low	Low	Low
Damen et al., 2021 (34)	Low	Low	Low	Low	Low	Low
Guastella et al., 2023 (35)	Low	Low	Low	Low	Low	Low
Guastella et al., 2015 (36)	Some concerns	Low	Low	Low	Low	Low
Le et al., 2022 (37)	Low	Low	Low	Low	Low	Low
Parker et al., 2017 (13)	Low	Low	Low	Low	Low	Low
Daniels et al., 2023 (38)	Low	Low	Low	Low	Some concerns	Some concerns
Bernaerts et al., 2020 (39)	Low	Low	Low	Low	Low	Low
Kong et al., 2021 (40)	Low	Low	Low	Low	Low	Low
Fastman et al., 2021 (41)	Low	Low	Low	Low	Low	Low

**①** Bias arising from the randomization process.

**②** Bias due to deviations from the intended intervention.

**③** Bias due to missing outcome data.

**④** Bias in the measurement of the outcome.

**⑤** Bias in the selection of the reported result.

### 3.4 Meta-analysis

#### 3.4.1 The effect of OXT administration on social impairments

**Figure 2:**
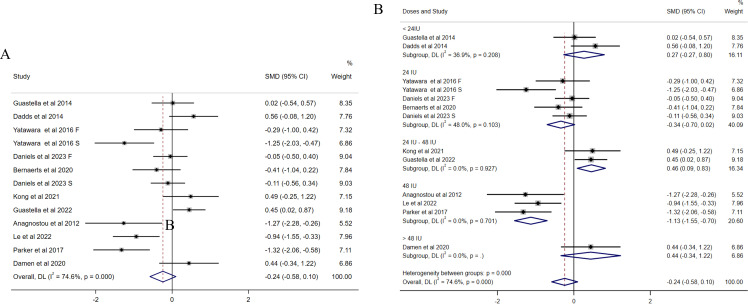
Forest plots of the effect of OXT on social impairments measured by the change in mean scores between baseline and posttreatment of total SRS scores. **(A)** Overall analysis. **(B)** Subgroup analysis depending on the dosage of OXT administration per day used among the included studies. Effect sizes are represented by filled points, the size of which indicates the weighting of the point estimate. 95% confidence intervals are represented by thin lines. The diamond represents the summary estimate (with the left/right edges indicating 95% confidence interval limits).

In terms of social impairments measured by total SRS scores, 11 studies (13, 31–40) were included (244 received OXT group and 236 received placebo). The effects of OXT on social impairments were examined, and the results suggested that OXT improved social impairments compared with placebo, whereas the difference did not reach significance. The results also indicated substantial heterogeneity among the included studies (I^2^ = 74.6%, **Figure 2A**). A sensitivity analysis was performed but did not identify the source of heterogeneity (**Supplementary Figure S1**), as previous studies suggested that OXT dosages might be a potential source of heterogeneity. OXT dosage significantly reduced the amount of residual heterogeneity, which highlights the importance of determining the most efficacious oxytocin dose (42). Due to the potential sources of heterogeneity caused by OXT dosage, and the different doses of OXT among the included studies (the dosages were reclassified into four groups as follows: <24 IU, 24 IU, 24 IU–48 IU, and >48 IU), subgroup analyses were conducted. The results showed that OXT dosage might be the source of heterogeneity. In addition, we observed that compared with placebo, the effect of OXT with the dosage of 48 IU per day on social impairments was significant [SMD = −1.13, 95% (−1.55, −0.70), **Figure 2B**]. The funnel plot (**Supplementary Figure S2**) and Egger’s test (p = 0.102) indicated that there was no publication bias among the included studies.

#### 3.4.2 The effect of OXT administration on repetitive behaviors

**Figure 3:**
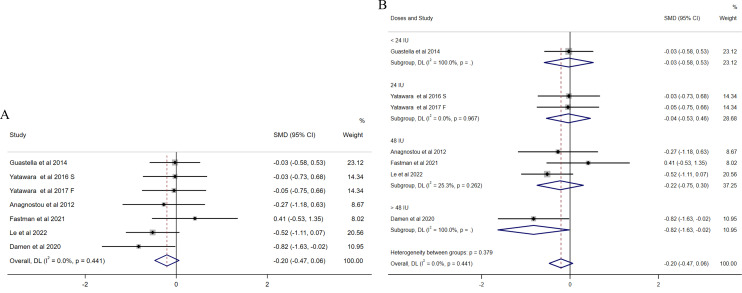
Forest plots of the effect of OXT on repetitive behaviors measured by the change in mean scores between baseline and posttreatment of RBS scores. **(A)** Overall analysis. **(B)** Subgroup analysis depending on the dosage of OXT administration per day used among the included studies (<24 IU, 24 IU, 48 IU, and >48 IU). Effect sizes are represented by filled points, the size of which indicates the weighting of the point estimate. 95% confidence intervals are represented by thin lines. The diamond represents the summary estimate (with the left/right edges indicating 95% confidence interval limits).

For repetitive behaviors, six studies (31, 32, 34, 36, 37, 41) focused on the mean changes in repetitive behaviors measured by RBS (**Figure 3**). No significant effect of intranasal OXT on repetitive behaviors was found in an initial overall analysis (SMD = −0.20, 95% CI (−0.47, 0.06), **Figure 3A**) and no presence of heterogeneity (I^2^ = 0%). Since the variety of intranasal OXT doses, the subgroup analysis based on daily total dose was calculated [the dosages were reclassified into four groups as follows: < 24 IU, 24 IU, 48 IU, and >48 IU)]. The results revealed non-significant results for all doses administrated, except the dose over 48 IU (SMD = −0.82, 95% CI (−1.63, −0.02), **Figure 3B**), while it was only assessed with one study. There was no publication bias among the included studies illustrated by the funnel plot (**Supplementary Figure S3**) and Egger’s test (p = 0.805).

### 3.5 Dose-response meta-analysis

**Figure 4:**
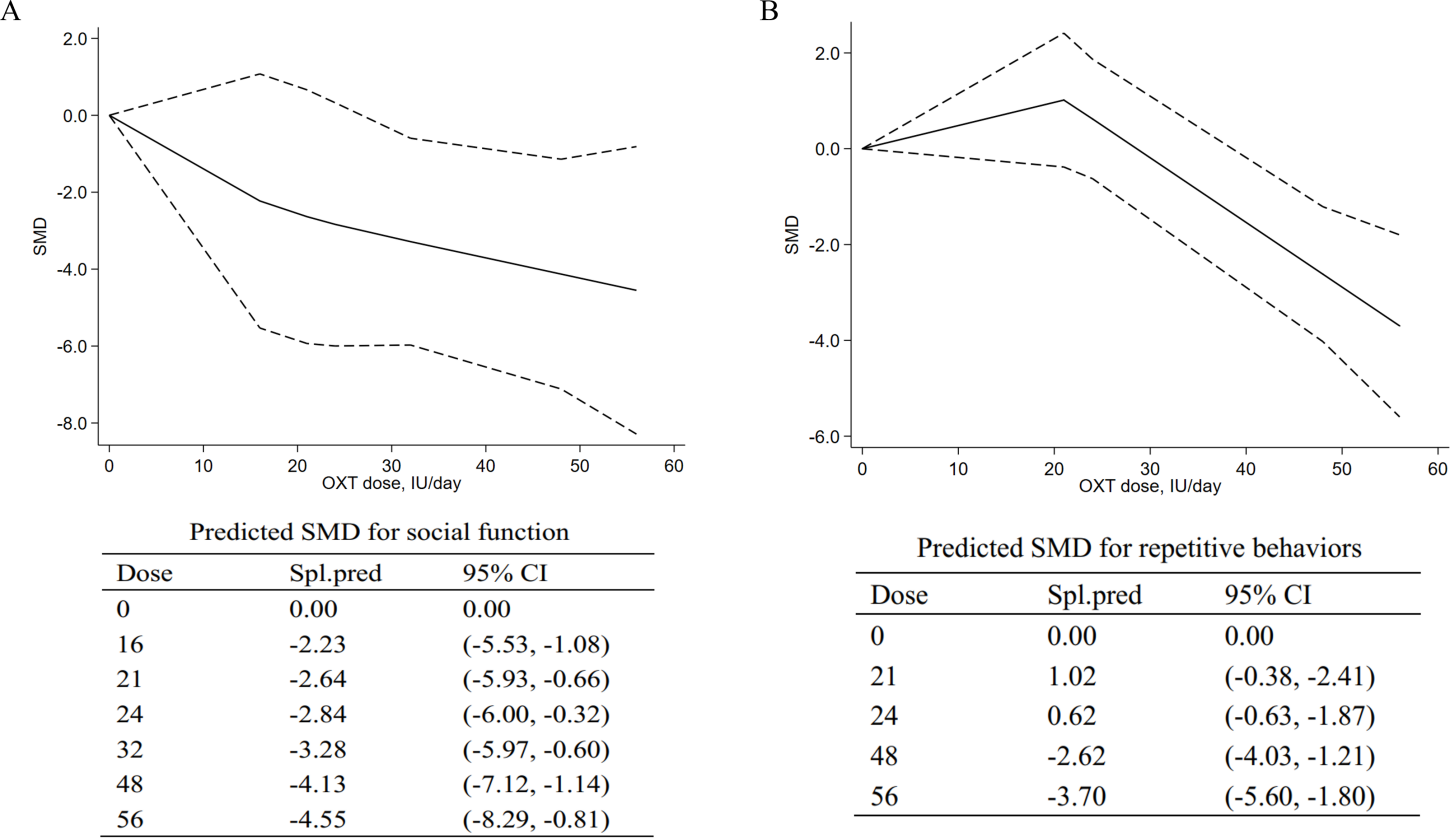
Dose–response meta-analyses for the relationship between oxytocin dose, social impairments, and repetitive behaviors. **(A)** Social impairments. **(B)** Repetitive behaviors. SMD, standardized mean difference; Spl.pred, spline model prediction. Solid lines represent dose–response associations between doses of OXT in IU/day and the mean change in social impairments **(A)** and repetitive behaviors **(B)**. Dashed lines represent the 95% confidence intervals for the spline model. Predicted standardized mean difference for negative and positive symptoms are reported below the figure in a table.

The OXT dose is modeled with restricted cubic splines in a random-effects model. In dose–response figures (**Figure 4**), a solid line represents the observed SMD changes, and for the spline model, the dashed lines are the 95% confidence intervals.

#### 3.5.1 Dose–response meta-analysis for social impairments

A dose–response meta-analysis was conducted to further explore the dose-dependent relationship of intranasal OXT with social impairment. The dose–response relationship between doses of OXT (18 IU/day to 56 IU/day) and the mean and standard deviation changes of the total SRS scores with the non-linear model were assessed. The model suggested that efficacy increased from 16 IU to 56 IU [16 IU, SMD = −2.23, 95% CI (−5.53, −1.08); 56 IU, SMD = −4.55, 95% CI (−8.29, −0.81)], indicating that higher doses could be more effective for social impairments in ASD. The pooled predicted dose–response curve and the mean differences of the models are provided in **Figure 4A**.

#### 3.5.2 Dose–response meta-analysis for repetitive behaviors

For repetitive behaviors, an inverted curve was observed for the model-based prediction (**Figure 4B**). For doses from 0 to 21 IU, the results suggested the decreasing effects on repetitive behaviors [21 IU, SMD = 1.02, 95% CI (−0.38, −2.41)]. For doses of 24 IU to 56 IU, the model suggested increasing effects on repetitive behaviors [24 IU, SMD = 0.62, 95% CI (−0.63, −1.87); 56 IU, SMD = −3.70, 95% CI (−5.62, −2.41)]. At higher doses, the curve was still in a downward direction, suggesting that higher doses may be more effective in treating repetitive behaviors.

## 4 Discussion

This meta-analysis of intervention trials was the first to address the limitations of previous reviews. It assesses the dose-dependent effect of OXT on social impairments and repetitive behaviors in ASD patients, with the application of methodologies not previously used in similar studies. These methods include the dose–response analysis to determine the optimal dosage for the improvement of social impairment and repetitive behaviors. Basic and clinical research in humans shows evidence that OXT administration delivers OXT directly from the nose to the brain, where it acts on central OXT receptors to produce its behavioral effects (43–45). In this context, the roles of OXT dosage and frequency of administration are of high interest. The results from the dose–response meta-analysis suggested that increased OXT administration per day could improve social impairments and reduce repetitive behavior, with higher doses of OXT being more efficacious for social impairments and repetitive behaviors.

In the current meta-analysis, we did not observe an overall beneficial effect of OXT administration on social impairments. A significant improvement in social impairments was found for the 48 IU per day subgroup. However, this was not observed in the highest group (> 48 IU), which might be due to the limited number of the included studies (only one study was estimated). The results of the dose–response meta-analysis suggested that the effect of OXT administration increases with high doses of OXT. In contrast to previous meta-analyses (46) that reported the absence of efficacy of OXT administration on social impairments, the current meta-analysis suggested a potential trend-level effect on social impairments of high-dose OXT. There were several explanations for the differences observed. First, social impairments were assessed diversely and differently from the current meta-analysis. In the previous meta-analysis conducted by Ooi et al. (2017) (46), social impairments assessed by emotion recognition, face/gaze processing, comprehension of affective speech, and theory of mind was pooled for meta-analysis, while the current meta-analysis only included RCTs that used total SRS scores to measure social impairments. The variety of measures for social impairments might contribute to the failure to detect the significant effect of OXT treatment in previous trials. Second, Ooi et al. (2017) (46) investigated the effect of OXT on social impairments with mixed doses administrated in ASD, which might be a failure to detect the therapeutic effects of ASD. Discrepancies between reports of single-dose and continuous treatment with different doses and frequencies were observed in previous studies; these factors can directly influence detecting the effects of OXT treatment. However, in the latest meta-analysis conducted with the multilevel meta-analytic model (14), the findings suggested that OXT does improve social impairments across a variety of measures such as eye gaze, emotion recognition, and prosocial behaviors [for details, see Huang et al. (2021) (14)]. A newly published meta-analysis using recently developed methods that account for publication bias and that can evaluate evidence for null models revealed that OXT doses would be significant factors affecting social outcomes (17). Notably, our current updated meta-analysis not only considered the frequency and dose of OXT administrated per day but also used the same measure (SRS) for social impairments. This allows us to assess the effect of OXT on social impairments more strictly regardless of heterogeneity caused by OXT doses and frequencies. It extends the previous analysis with different meta-analytic models to verify and clarify the role of OXT as an effective intervention in ASD.

Although a limited number of studies reported RBS scores regarding repetitive behaviors in ASD, Higher doses of OXT may be more effective for repetitive behaviors, according to our dose–response model. The previous meta-analysis (Ooi et al. (2017) (46) did not report a significant effect on restricted, repetitive behavior, depending on the four studies included (46). Moreover, a lack of efficacy of OXT treatment was found in meta-analytic estimates for non-social domains (14) including restricted and repetitive behaviors, obsessive-compulsive disorder, and depression. Although dose–response results for repetitive behaviors are less clear with a limited number of studies, higher doses may be beneficial. These effects on repetitiveness observed in the current study are consistent with previous studies in ASD that have shown beneficial effects of OXT on repetitiveness after 4 hours of OXT (47) and after 6 weeks of daily administration (11, 31). However, no improvement in repetitive behaviors was also observed after 4 or 5 days or 8 weeks of OXT administration (13, 36). Moreover, different measurements of the changed repetitive behaviors after OXT administration would have an impact on the beneficial effect of OXT. In detail, some studies adopted assessments based on self-reports [i.e. Anagnostou et al. (2012) (31)], while other studies adopted informant-based reports of repetitive behaviors (13, 33, 36). Taken together, these findings may therefore be an indication that the duration of treatment, the timing of measurement after OXT administration, and the measurement of outcomes have an impact on the effects of OXT treatment on repetitive behaviors.

Certain limitations of our study and existing RCTs provide caveats to consider and, if possible, investigate in future studies before drawing firm conclusions. First, our small sample size limits the ability to draw definitive conclusions about the efficacy of OXT for ASD symptoms, especially in social symptoms and repetitive behaviors based on the results of this study. Second, the relatively wide age range, pubertal status, and baseline levels of OXT were also not taken into account in our current meta-analysis, which might affect the robustness of the current results. Third, different outcome measurements (self-reports and informant-based reports of repetitive behaviors) are included to pool the meta-analysis. Individual differences in baseline hormone levels can influence the effects of OXT. Fourth, a sex-specific effect of OT administration on ASD symptoms did not take into account due the limited data among the included studies.

## 5 Conclusions

The optimal dose of OXT for ASD is currently being debated (38, 48, 49), and there is increasing evidence of an inverse U dose–response curve for OXT in subclinical and clinical trials (15, 50). However, it is worth noticing that the results of preclinical studies support the pharmacodynamic advantage of administering a larger dose (51, 52), which partly supports our results. Although the dose–response meta-analysis does not allow firm conclusions to be drawn, it suggests that high doses of intranasal OXT may be more effective for both social symptoms and repetitive behaviors. Standardization dosage, dose frequency, and treatment duration of OXT administration are suggested as essential for future human trials and clinical applications of OXT.

## Data Availability

The original contributions presented in the study are included in the article/**Supplementary Material**. Further inquiries can be directed to the corresponding author.
